# Editorial Department

**Published:** 1853-09

**Authors:** 


*August* 11, 1853.

Dear Sir,—You will confer a favor on me, and some others, by stating through the Buffalo Medical Journal, your opinion of the propriety of using opium, with, or without astringents, in the *first stage of dysentery*, and also whether rhubarb is considered a good cathartic in the disease.

Yours, &c., MEDICUS.

With reference to the use of opium in dysentery, we would refer our correspondent to remarks on the management of that disease which will appear in our next issue. We would simply say here, that, in our opinion, opium may be given without impropriety, and often with signal advantage, sometimes, indeed, apparently arresting the disease, in the first stage of the affection—the earlier the better.

As respects rhubarb, we have frequently prescribed it as a laxative during convalescence from dysentery, and consider it well suited to that stage of the disease. If, however, we desire an effective cathartic operation in the early part, or during the progress of the disease, the saline cathartics seem to us preferable, unless there are peculiar reasons for apprehension in consequence of their hydragogue property. We have supposed them to be inappropriate, on this account, during the prevalence of epidemic cholera.

